# Variations of the Transverse Sinus: Review with an Unusual Case Report

**DOI:** 10.7759/cureus.3248

**Published:** 2018-09-04

**Authors:** Chrissie Massrey, Juan J Altafulla, Joe Iwanaga, Zachary Litvack, Basem Ishak, Rod J Oskouian, Marios Loukas, R. Shane Tubbs

**Affiliations:** 1 Seattle Science Foundation, Seattle, USA; 2 Swedish Neuroscience Institute, Seattle Science Foundation, Seattle, USA; 3 Medical Education and Simulation, Seattle Science Foundation, Seattle, USA; 4 Neurosurgery, Swedish Neuroscience Institute, Seattle, USA; 5 Neurosurgery, Seattle Science Foundation, Seattle, USA; 6 Anatomical Sciences, St. George's University, St. George's, GRD

**Keywords:** transverse sinus, dural venous sinuses, anatomy, variation, fenestration

## Abstract

The dural venous sinuses are venous channels in the cranium that drain blood and cerebrospinal fluid circulating from the brain into the vascular system via the internal jugular veins. The transverse sinus is a dural venous sinus present in the posterior aspect of the cranium. We report an unusual variant of this sinus with the presence of a fenestration at its proximal segment. We will review and discuss the background and the potential clinical relevance of this anatomical variation.

## Introduction

Blood drains from the brain and the cranium via a network of venous channels termed the dural venous sinuses. The dural venous sinuses are unlike systemic veins in that they do not have valves or musculature, however, they are similar to systemic veins in that they are lined with endothelium. They are present between the endosteal and meningeal layers of the dura mater [1]. The major dural venous sinuses in the cranium include the superior and inferior sagittal sinuses, the straight, transverse, sigmoid, occipital, cavernous, intercavernous, superior and inferior petrosal, sphenoparietal, and marginal sinuses and basilar plexus [2].

The transverse sinuses begin at the torcular Herophili, which is the connection of the superior sagittal, straight, and occipital sinuses. The right transverse sinus is usually a continuation of the superior sagittal sinus, draining blood from the superficial structures of the brain and is typically larger than the left transverse sinus, and the left is usually continuous with the straight sinus. The transverse sinuses course in the attached margin of the tentorium cerebelli, receiving blood from the lateral temporal surface and the basal surface of the temporal and occipital lobes, ultimately reaching the posterolateral part of the petrous part of the temporal bone to drain into the sigmoid sinus, which will then drain into the internal jugular vein [2].

As the embryo enlarges, the transverse sinus will also enlarge up until six to seven months of gestation. Variations of the transverse sinus such as irregular diameters and margins, septum formations, and missing segments, might occur if the transverse sinus rapidly increases and decreases in size during development. After birth, the diameter of the transverse sinuses decreases slightly and becomes adult-sized at about one year of age [3]. Additionally, in a study by Okudera et al., ballooning of the transverse sinus lateral to medial may also occur beginning at about four months of gestation. Irregular ballooning and shrinkage of the sinus may result in the formation of septa, fenestrations, or missing segments of the transverse sinus [4-5].

In this paper, we discuss the transverse sinus and its variations and report an unusual case.

## Case presentation

**Figure 1:**
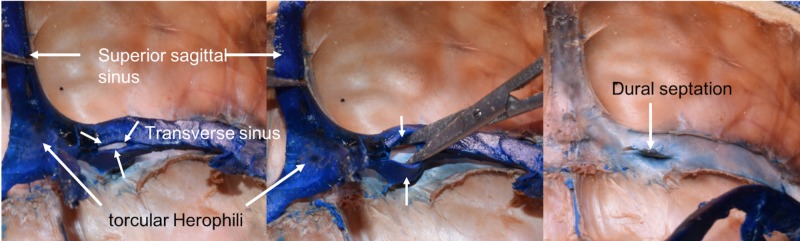
Left: transverse sinus with proximal fenestration (short arrows); Middle: widening of fenestration (arrows) with scissors; Right: Following removal/retraction of blue latex from within the left transverse sinus, note the intraluminal dural septation.

During the routine dissection of the cranium of an adult male cadaver aged 76 years at death, an unusual left transverse sinus was identified. The specimen had previously undergone blue latex injection of the left and right internal jugular veins. Following removal of the brain, the venous sinuses of the posterior cranial fossa were dissected. Noteworthy, the left transverse sinus was fenestrated at its most proximal part approximately 1 cm lateral to the torcular Herophili (Figure 1). At this point, the transverse sinus was split into two more or less equal parts. Distal to the fenestrated sinus, the transverse sinus reunited and traveled in normal fashion to its drainage into the left sigmoid sinus. When the blue latex that filled the transverse sinus was retracted, a clear dural septum was noted within the lumen of the fenestrated part of the sinus (Figure 1). No other anatomical variations and specifically of the dural venous sinuses were noted on left or right sides. The left and right transverse sinuses were approximately the same size, and the superior sagittal sinus drained primarily into the right transverse sinus. The straight sinus drained preferentially into the left transverse sinus proximal to its fenestrated segment.

## Discussion

It is important to note the anatomical variations that may be present are not mistaken for pathology. Our case report is a rare anatomical variation of the transverse sinus; that variant being a fenestrated segment. Browder and Kaplan (1976) reported many variations in the transverse sinuses [6]. Perhaps one of the most significant to note is the complete absence of the transverse sinus [7]. Browder and Kaplan also reported a doubling of the distal 1-3 cm of the superior sagittal sinus, giving rise to a small transverse sinus. They also found cases with the presence of a vascular mesh within the transverse sinus, and they identified cases of transverse sinuses consisting of many connections to the torcular Herophili. In all of these variations, the transverse sinus was typically smaller than normal on the side with the variation [6].

In addition to these variations, aplasia or hypoplasia of the transverse sinus [6, 8]. Bergman et al. [9] reported a variation in the transverse sinus in which the proximal part was missing, and Knott [10], in 1881, noted two cases in which there was a complete absence of the right transverse sinus. Additionally, Furstenberg [11] reported a variation in which the sigmoid sinus was absent and the transverse sinus was present outside the skull. Septation of the transverse sinus may occur in infants that have overlapping lambdoid sutures [12]. Often, a septum within the transverse sinus as seen in the present case report can imitate the appearance of thrombus or arachnoid granulations on venography [13].

Goyal et al. [14] performed a retrospective study on adults to see the anatomical variations in the dural venous sinuses using MR venography. They noticed the transverse sinus to be symmetrical in 66.9% of their patients. In the other third, the left transverse sinus was hypoplastic in 21.3% and aplastic in 4.1%, while the right side was hypoplastic in 5.5% of patients and aplastic in the remaining 0.7%. A total of 1.6% patients had bilateral hypoplastic transverse sinuses. Conversely, in a study by Alper et al. [15] symmetrical transverse sinuses were seen in 31% of the cases. A hypoplastic left transverse sinus was seen in 39% and the sinus was aplastic in 20% of the patients. A hypoplastic right transverse sinus was seen in 6% and was aplastic in 4% of cases.

Because hypoplasia or aplasia of the left transverse sinus is more common than on the right, the right jugular system will more often have an increased capacity. Bayarogullari et al. [16] reported that the superior sagittal sinus more commonly drained into the right transverse sinus, whereas the straight sinus more often drained into the left transverse sinus. They also found, in 20 of their cases, another variation in which the superior sagittal sinus bifurcated. In two of the cases that had a bifurcated superior sagittal sinus, the straight sinus drained into the distal right transverse sinus and actually formed an accessory transverse sinus [16].

## Conclusions

Variation of the transverse sinuses is not uncommon and should be considered before any pathology, such as venous sinus thrombosis, is diagnosed. Fenestrations of this dural venous sinus as seen in the case reported herein can occur but reports in the literature are scant.
